# TRAINING OF CHILDREN’S AND ADOLESCENTS’ FAMILY MEMBERS IN HOME PARENTERAL NUTRITION CARE

**DOI:** 10.1590/1984-0462/;2019;37;3;00002

**Published:** 2019-05-09

**Authors:** Maria Carolina Witkowski, Rosiani de Souza Silveira, Daiane Marques Durant, Alessandra Cortes de Carvalho, Daltro Luiz Alves Nunes, Marcia Camaratta Anton, Myriam Fonte Marques, Silvana Maria Zarth, Helena Becker Issi, Helena Ayako Sueno Goldani

**Affiliations:** a Hospital de Clínicas de Porto Alegre, Porto Alegre, RS, Brazil.; b Universidade Federal do Rio Grande do Sul, Porto Alegre, RS, Brazil.

**Keywords:** Parenteral nutrition, home, Training, Child, Pediatric nursing, Caregivers, Nutrição parenteral no domicílio, Capacitação, Criança, Enfermagem pediátrica, Cuidadores

## Abstract

**Objective:**

To report the experience of the training in home parenteral nutrition (PN) directed to family members of children and adolescents participating in a multidisciplinary intestinal rehabilitation program of a tertiary public hospital.

**Methods:**

Cross-sectional descriptive study with family caregivers of patients from the Intestinal Rehabilitation Program of Hospital de Clínicas de Porto Alegre, RS, Brazil, from July/2014 to January/2017. Inclusion criteria: family members of children aged 30 days to 17 years and estimated PN use ≥8 weeks; and family members motivated to care for the child. The training covered: hand washing and disinfection; infusion pump handling; and central venous catheter (CVC) and PN care. Outcomes assessed: catheter-related bloodstream infection (CRBSI) rate, accidental CVC exit, end of PN infusion with more than 60minutes of delay or advance compared to the time predicted, mechanical obstruction, bleeding in the CVC insertion site, and death.

**Results:**

Twenty-seven family members of 17 children were trained. Their median age was 28 (18-60) years, and 63% were mothers. The mean CRBSI rate was 1.7/1,000 days of CVC use, and 29.4% of patients had at least one episode of accidental CVC exit. There were no complications related to PN infusion, bleeding, or death.

**Conclusions:**

The training of family caregivers allowed the safe implementation of home PN, with the active participation of families, making the procedure feasible in the public health system in Brazil.

## RESUMO

**Objetivo:**

Apresentar a experiência da capacitação de familiares de crianças e adolescentes participantes de um programa multiprofissional de reabilitação intestinal de um hospital público terciário para uso de nutrição parenteral (NP) no domicílio.

**Métodos:**

Estudo descritivo transversal com familiares cuidadores de pacientes do Programa de Reabilitação Intestinal do Hospital de Clínicas de Porto Alegre, RS, Brasil, entre julho/2014 e janeiro/2017. Critérios de inclusão: familiares das crianças com idades entre 30dias e 17 anos e previsão de uso de NP≥8 semanas; e familiares que demonstrassem motivação para os cuidados da criança. Acapacitação contemplou: lavagem e higienização das mãos; manuseio da bomba de infusão; cuidados com cateter venoso central (CVC) e com solução de NP. Desfechos avaliados foram: taxa de infecção de corrente sanguínea relacionada ao CVC (ICSRC), saída acidental do CVC, término da infusão da NP com atraso ou adiantamento maior que 60minutos em comparação ao previsto, obstrução mecânica, sangramento do sítio de inserção do CVC e óbito.

**Resultados:**

Foram capacitados 27 familiares de 17 crianças, com mediana de idade de 28 (18-60) anos, dos quais 63% eram mães. A taxa média de ICSRC observada foi 1,7/1.000 dias de uso de CVC, e a saída acidental do CVC ocorreu em 29,4% dos pacientes. Não foram observadas complicações referentes à infusão da NP, a sangramento ou a óbito.

**Conclusões:**

A capacitação de familiares cuidadores possibilitou a execução de maneira segura da NP no domicílio com participação ativa das famílias, tornando o procedimento viável no sistema público de saúde no Brasil.

## INTRODUCTION

Intestinal failure (IF) is a severe malabsorption condition that demands artificial nutrition via parenteral.^1^ It can be acute - when the patient depends on parenteral nutrition (PN) for up to 90 days -,^2^ or chronic - when the time of PN use exceeds 90 days. The first treatment option for patients who need long-term PN is home PN.^3^

Home PN allows both patients and their family to live outside the hospital. When performed at home, PN can not only improve the quality of life of patients and their entire family, but also increase survival, promote social interaction, and reduce costs with health.^4^ Home PN was introduced in Europe in the decade of 1970,^5^ and requires an integrated multidisciplinary team of physicians, nurses, pharmacists, nutritionists, psychologists, and social workers.^6^ ^,^ ^7^

Patients on home PN demand specific long-term care and dedication from family members trained to care for children and adolescents after hospital discharge. Some prerequisites are essential for the child to go home safely, leading to the success of intestinal rehabilitation.^8^ For instance, family caregivers must be motivated to care for the patient at home and capable of dealing with clinical, emotional, and technical issues related to home PN.^9^

Due to the complexity of home PN infusion care in children and adolescents, training in techniques and care to handle catheters, infusions, and equipment is crucial to avoid complications. The most common complication in this group of patients is catheter-related bloodstream infection (CRBSI), which can lead to a mortality rate of 12 to 25%.^10^ Other possible complications are accidental catheter exit, obstruction, and bleeding.^11^

Although home PN with the active participation of families of children and adolescents is a technique validated in Europe, there is no systematic record of this practice in the Brazilian public health system. Thus, this study aimed to report the experience of the training in home PN directed to family members of children and adolescents participating in a multidisciplinary intestinal rehabilitation program of a tertiary public hospital.

## METHOD

The Research Ethics Committee of Hospital de Clínicas de Porto Alegre, RS, Brazil, approved this study under the CAAE protocol No. 21748119.2.0000.5327 and GPPG 13-0383. Parentsand guardians of the children were informed about the goals of the research and signed the informed consent form.

This cross-sectional descriptive study was conducted with family caregivers of children and adolescents participating in a Multidisciplinary Intestinal Rehabilitation Program for Children and Adolescents of Hospital de Clínicas de Porto Alegre (PRICA-HCPA), created in January 2014. The program has a multidisciplinary team comprising pediatric gastroenterologists, pediatric surgeons, physician nutrition specialists, nurses, pharmacists, nutritionists, psychologists, social workers, and administrative staff. The program provides care to long-term PN-dependent patients and enables their return home. In this scenario, PRICA-HCPA developed a formal training guideline applied by nurses and directed to close family caregivers responsible for the direct care of these patients.

Inclusion criteria to participate in the study were:

- Family members of children aged 30 days to incomplete 18 years.
- Family members of children who used PN for at least eight weeks.
- Family members willing and motivated to care for the child at home.

After formalizing the desire for discharge with PN, family members were evaluated in their manual skills and psychosocial and socio-environmental conditions by a nurse, psychologist, and social worker, respectively. The following minimum socio-environmental criteria assessed for discharge and home PN were: household location, in urban or rural areas; patient’s access to the local health service, as well as of local health teams to the household; presence of sewage system, tap water, electrical energy, and hygiene care in the environment.

Family caregivers participating in the study were consecutively added, following the inclusion of patients in ­PRICA-HCPA. Data were collected from July 2014 to January 2017. The multidisciplinary team monitored the patients during the follow-up period of the program, and the researchers collected data at the end of the study time. The follow-up period for each family member was the time in which the patient was on home PN until the completion of the research. A specific questionnaire provided the demographic data of family caregivers regarding marital status, schooling, and household income according to minimum wage.

Researchers developed an instrument to train family caregivers, covering the following items: training for hand washing and disinfection, infusion pump handling, and CVC and PN care. When patients also needed enteral nutrition, family caregivers were trained in care related to the administration of nasogastric tube or gastrostomy feeding. Thenurses of the program gave all training in theory and practice, regardless of the children’s age group, and filled the training instrument. The total training period for each caregiver should be at least 15 days.

**Chart 1:**
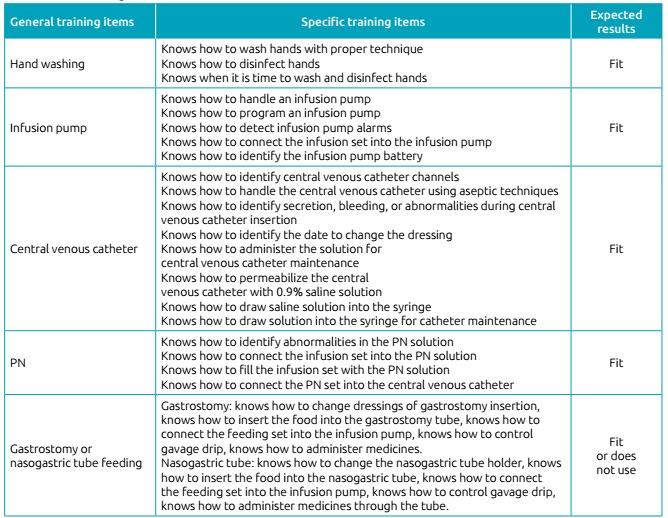
Theoretical and practical content covered in the training of family caregivers of children on home parenteral nutrition, Porto Alegre, RS, 2017.

Additional information related to patients was collected from electronic medical records*.* Training started after the family members agreed to participate in the program. Chart1 shows the items used to train each family member.

PN: parenteral nutrition.

At the end of 15 days of theoretical and practical training, family members were formally assessed on their ability to perform all procedures related to patient care. After this period, family members who did not reach full qualification in all necessary items underwent new training in the lacking items to complete their instruction. The nurses participating in the PRICA-HCPA carefully conducted the training process and assessment on the technical ability of each family caregiver. All family caregivers received a full training certificate of home PN care.

In addition to the training of family caregivers, home care nurses from the respective cities were also trained prior to hospital discharge. This training lasted 2 to 4 hours with a qualification certificate. After the training of all caregiver teams (family members and ambulatory home care teams), the patient was discharged.

After hospital discharge, patients and their families stayed for at least two days at Casa de Apoio of HCPA (a nonprofit organization that provides social services and accommodation to users of the public health system), where PRICA-HCPA nurses supervised them and simulated home care. Next, patients went home with the training unit (PRICA-HCPA nurses) and trained people (family members and ambulatory home care nurses) sharing the responsibility for their care.

Outside the hospital, family caregivers installed the PN infusion system, always under the daily supervision and educational monitoring of PRICA-HCPA nurses when at Casa de Apoio or home care nurses from the city when at home. After at least two months of daily supervision at home, some family caregivers were evaluated in their autonomy regarding home PN care and took over full care, with weekly visits from home care teams. The PN infusion usually lasted a period of 12 to 14hours. Patients who needed 24-hour PN infusions required at least two trained family members for discharge. The PN solution was customized for each patient, prepared by a specialized pharmaceutical company, delivered to the patient’s home, and stored at optimal temperature.

This study used the JMS infusion pump OT711^®^, Med-Tech Inc., Japan, which is easy to handle. The pumps were checked and calibrated previously according to the clinical engineering protocol of the institution. PRICA-HCPA provided the materials needed, such as infusion pumps, infusion sets, and other health care materials.

We assessed the following outcomes related to home PN complications: end of PN with more than 60minutes of delay or advance compared to the time predicted, CRBSI rate calculated per 1,000 days of CVC use, mechanical CVC obstruction, accidental CVC exit, bleeding in the CVC insertion site, and death. This information was gathered from the electronic medical records of patients at the end of data collection.

We entered the data into the database of the Statistical Package for the Social Sciences(SPSS) version 18.0 (SPSS Inc., Chicago, IL, USA), with double entry to confirm the records. Data were analyzed using descriptive statistics and expressed by mean, and absolute and relative frequency. We used the Mann-Whitney U test to check for associations of the number of episodes of accidental CVC exit with patient’s age and time of PN use. We considered p<0.05 significant.

## RESULTS

A total of 27 family caregivers of 17 patients on PN were eligible for the study. All 27 family members participated in the study, constituting the sample analyzed. Table 1 shows the general characteristics of the sample.

**Table 1:** Sociodemographic characteristics of 27 family caregivers trained for home care of 17 children and adolescents participating in the Intestinal Rehabilitation Program for Children and Adolescents, Porto Alegre, RS, 2017.

Characteristics	n (%)
Females	21 (77.7)
Age (years)	
≤20	01 (3.7)
>20 ≤40	22 (81.5)
>40	04 (14.8)
Marital status (main caregivers of each child)	
Married or lives with a partner	14 (51.8)
Single	3 (11.1)
Widower	2 (7.4)
Divorced, separated	8 (29.7)
Trained family member	
Mother	17 (63.0)
Father	6 (22.2)
Grandmother	2 (7.4)
Other (siblings or cousins)	2 (7.4)
Schooling (years)	
<5	3 (11.1)
≥5 <10	21 (77.8)
≥10	3 (11.1)
Household income according to minimum wage of 17 families	
<minimum wage	1 (5.9)
≥minimum wage and >two times the minimum wage	6 (35.3)
≥two times the minimum wage and >three times the minimum wage	8 (47.0)
≥three times the minimum wage	2 (11.8)
Income meets basic needs	14 (82.3)

The median age of family caregivers was 28 (18-60) years, with a prevalence of females. All family caregivers trained were literate. The minimum training period was 15 days, but 11 (40.7%) family members needed more time to finish the qualification. The median training time of family caregivers was 19 (15-45) days.

Family members of 17 children were trained. The median age of the patients was 12 (2-164) months; 14 (82.3%) were males; 11 (64.7%) had short bowel syndrome, and 6 (35.3%) had intestinal dysmotility. The median time of PN use during hospitalization was 6 (2-32) months. Six patients were rehabilitated and had the PN suspended before the end of the study.

Among the children included in the study, 41.2% (n=7) had more than one family caregiver qualified for home care.

Regarding complications in the children and adolescents on home PN (Table 2), we found a mean CRBSI rate of 1.7/1,000 days of CVC use. The most common CVC complication was the accidental catheter exit, in 29.4% (n=5) of patients. The median age of patients who had at least one episode of accidental CVC exit was 43 (40-110) months, while the median age of those who had no episodes was 8.5 (4-56) months. Patient’s age and time of PN use were related to these episodes. In the group of patients who had accidental CVC exit, the median age and time of PN use were higher, when compared to the group that had no episodes (p=0.006) (Table 3).

**Table 2:** Complications during home parenteral nutrition in 17 children and adolescents participating in the Intestinal Rehabilitation Program for Children and Adolescents, Porto Alegre, RS, 2017.

Complications	n (%)
Parenteral nutrition infusion	
Finished with more than 60minutes of delay or advance compared to the time predicted	0 (0.0)
Related to central venous catheter	
Infection rate	1.7^a^
Catheter lumen obstruction	1 (5.9)
Accidental exit	5 (29.4)
Bleeding	0 (0.0)
Death	0 (0.0)

^a^catheter-related bloodstream infection rate at home per 1,000 days of use, that is, 1.7/1,000 days of central venous catheter use=6 infections in 3,529 days of use.

**Table 3:** Relationship of patient’s age and time of parenteral nutrition use with the number of episodes of accidental catheter exit, Porto Alegre, RS, 2017.

	Accidental catheter exit		p-value^a^
	Yes	No	
Age - median (minimum-maximum)	43months (38-164)	8.5months (2-121)	0.006
PN use - median (minimum-maximum)	13months (11-30)	5.5months (2-18)	0.006

^a^Mann-Whitney U test; PN: parenteral nutrition.

## DISCUSSION

This study presented the results of the training of children’s and adolescents’ family caregivers in home PN care of patients participating in the intestinal rehabilitation program for children and adolescents of a public university hospital in Southern Brazil. It is a pioneering project in the country that sought to facilitate the practice of home PN for children and adolescents, with the active participation of family caregivers users of the public health system.

The literature has demonstrated that children with IF have benefited from treatments in reference intestinal rehabilitation centers with multidisciplinary teams,^12^ ^,^ ^13^ and has reports on home care practices carried out by children’s and adolescents’ parents or close family members. These practices include those performed with PN infusion, as well as feeding and medication infusions via nasogastric tube or gastrostomy.^7^ ^,^ ^14^

Home PN was indicated as a safe practice, well established in both children and adults.^7^ ^,^ ^13^ ^,^ ^15^ The training of children’s family members for at least two to three weeks before hospital discharge showed good results in European countries,^16^ ^,^ ^17^ ^,^ ^18^ even when it covered the addition of electrolyte solutions to the PN bag.^16^ The training also improved the transition from hospital to home care.^19^ In our study, the families of children and adolescents on home PN were trained for at least two weeks and received ready-to-use PN infusion bags, with no need to prepare or administer other solutions in the bag before infusion.

In addition to CVC handling techniques and PN infusion care, social and psychological factors are important during the assessment prior to hospital discharge. Although each patient should have two trained family caregivers,^14^most patients in this study had only one, and additional care related to social support and home nursing were provided. Divorced parents who shared the care of the child had both to be trained in all required items, and participate in the care at least once a week to not forget the skills learned.^5^ ^,^ ^18^ Most trained family members in this study were mothers, and, among them, the majority was married or lived with a partner.

The social and psychological evaluation is also important after hospital discharge. In this study, 82.3% of the qualified family members declared that their income meets basic needs. We found different results in another study, in which the income did not meet basic needs, revealing a decreased workload, reduced quality of life, and increased depression among family members more involved in home care.^20^

Some complications related to PN handling and infusion can occur during the patient’s stay at home. Patients were remotely monitored using a telephone number to on-call physicians and nurses. Patients called this number and received appropriate instructions, including if they needed to visit the emergency department.

In this study, no patient finished PN with more than 60minutes of delay or advance compared to the time predicted, had a bleeding episode, or died at home. Regarding infection rates, a systematic review analyzed 39 studies from 14 countries, and the CRBSI rate ranged from 0.38 to 4.58/1,000 days of CVC use.^10^ The present study had a mean CRBSI rate of 1.7/1,000 days. This rate is within the variations described and near the desired goal - less than 1/1,000 days of CVC use.^21^

Only one patient (5.9%) had CVC lumen obstruction- a frequent complication that can occur in 2 to 75% of patients on long-term PN -;^22^ ^,^ ^23^ however, five (29.4%) had episodes of accidental CVC exit. This high frequency was caused by the patient’s return to social interaction, including physical and leisure activities. In the literature, the first CVC-related complications happen on average after 180days of home care.^24^

In the present study, the median time for accidental catheter exit to occur was 13 (11-30) months. No patient had bleedings in the CVC insertion site or died at home. A recent multicenter study carried out in the Czech Republic revealed the results of 66 pediatric patients, with a mortality rate of 19.7% and infection rate of 1.58/1,000 days of CVC use.^25^

The purpose of training family caregivers is to provide safe hospital discharge, allowing continuous and planned care according to patient needs. It is crucial that family caregivers recognize complications for early intervention. The knowledge about how to identify signs and symptoms related to complications must be acquired during training.^26^

A limitation of the study was not assessing the quality of life as an indicator of patients’ and family members’ well-being. A research showed that the quality of life of patients and family members improved after hospital discharge with home PN.^26^ Another limitation could be the number of participants in the study. However, for the first time in Brazil, the formal training of family caregivers and their active participation in home PN care was presented.

This study provides elements for the development of a baseline home PN care in Brazil for patients users of the public health system. Changes are necessary since the current legislation indicates that this practice is the sole responsibility of the nurse.^27^ In this regard, we present the results of the implementation of home PN, which showed no severe adverse events, and had active participation of families trained and supervised by nurses.

In conclusion, this study demonstrated that the discharge process for children and adolescents on PN could be feasible, safe, and effective with training in home PN care targeted at family caregivers of children. Home PN practices in children and adolescents must be improved to promote a better clinical condition for patients and quality of life for their family members and caregivers.
